# Screening and evolution of a novel protist xylose isomerase from the termite *Reticulitermes speratus* for efficient xylose fermentation in *Saccharomyces cerevisiae*

**DOI:** 10.1186/s13068-017-0890-1

**Published:** 2017-08-23

**Authors:** Satoshi Katahira, Nobuhiko Muramoto, Shigeharu Moriya, Risa Nagura, Nobuki Tada, Noriko Yasutani, Moriya Ohkuma, Toru Onishi, Kenro Tokuhiro

**Affiliations:** 1 Bioinspired Systems Research-Domain, Toyota Central R&D Labs., Inc., 41-1, Yokomichi, Nagakute, Aichi 480-1192 Japan; 2 RIKEN Center for Sustainable Resource Science, 1-7-22 Suehiro-cho, Tsurumi-ku, Yokohama, Kanagawa 230-0045 Japan; 3 Biotechnology and Afforestation Laboratory, New Business Planning Div, Toyota Motor Corporation, 1099 Marune, Kurozasa-cho, Miyoshi, Aichi 470-0201 Japan; 4 RIKEN Center for Sustainable Resource Science, 3-1-1 Koyadai, Tsukuba, Ibaraki 305-0074 Japan; 5 Japan Collection of Microorganisms, RIKEN BioResource Center, 3-1-1 Koyadai, Tsukuba, Ibaraki 305-0074 Japan

**Keywords:** Xylose isomerase, Termite, Cloning and functional expression of XI gene, *Saccharomyces cerevisiae*, Xylose fermentation, Ethanol production, Mutagenesis

## Abstract

**Background:**

The yeast *Saccharomyces cerevisiae*, a promising host for lignocellulosic bioethanol production, is unable to metabolize xylose. In attempts to confer xylose utilization ability in *S. cerevisiae*, a number of xylose isomerase (XI) genes have been expressed heterologously in this yeast. Although several of these XI encoding genes were functionally expressed in *S. cerevisiae*, the need still exists for a *S. cerevisiae* strain with improved xylose utilization ability for use in the commercial production of bioethanol. Although currently much effort has been devoted to achieve the objective, one of the solutions is to search for a new XI gene that would confer superior xylose utilization in *S. cerevisiae*. Here, we searched for novel XI genes from the protists residing in the hindgut of the termite *Reticulitermes speratus*.

**Results:**

Eight novel XI genes were obtained from a cDNA library, prepared from the protists of the *R. speratus* hindgut, by PCR amplification using degenerated primers based on highly conserved regions of amino acid sequences of different XIs. Phylogenetic analysis classified these cloned XIs into two groups, one showed relatively high similarities to *Bacteroidetes* and the other was comparatively similar to *Firmicutes*. The growth rate and the xylose consumption rate of the *S. cerevisiae* strain expressing the novel XI, which exhibited highest XI activity among the eight XIs, were superior to those exhibited by the strain expressing the XI gene from *Piromyces* sp. E2. Substitution of the asparagine residue at position 337 of the novel XI with a cysteine further improved the xylose utilization ability of the yeast strain. Interestingly, introducing point mutations in the corresponding asparagine residues in XIs originated from other organisms, such as *Piromyces* sp. E2 or *Clostridium phytofermentans*, similarly improved xylose utilization in *S. cerevisiae*.

**Conclusions:**

A novel XI gene conferring superior xylose utilization in *S. cerevisiae* was successfully isolated from the protists in the termite hindgut. Isolation of this XI gene and identification of the point mutation described in this study might contribute to improving the productivity of industrial bioethanol.

**Electronic supplementary material:**

The online version of this article (doi:10.1186/s13068-017-0890-1) contains supplementary material, which is available to authorized users.

## Background

Bioethanol produced from regenerative biomass resources is a promising sustainable alternative to fossil fuels in order to reduce greenhouse gas emitted from the automobiles. Because of increasing global public concern about food security and environment, it is preferable to use lignocellulosic biomass, instead of edible sugar, as the substrate for bioethanol fermentation. Lignocellulosic biomass, such as corn stover, wheat straw, and rice straw among others, is an attractive substrate because it is cheap and abundantly found as agricultural and forest residues.

Yeast *Saccharomyces cerevisiae* is widely used as a candidate host organism for bioethanol production due to its robustness, inhibitor tolerance, and high ethanol productivity [1]. However, to obtain the highest ethanol yield from each biomass substrate, co-fermentation of all of sugars contained in the lignocellulosic biomass hydrolysate is necessary. Since wild-type *S. cerevisiae* strains cannot utilize d-xylose, the second major sugar present in lignocellulosic hydrolysate (up to 40%), numerous attempts have been made to recombinantly create yeast strains that can efficiently ferment xylose [2–6]. Two different pathways have been proposed for the anaerobic fermentation of xylose by *S. cerevisiae*. The first pathway consists of sequential redox reaction of xylose, catalyzed by xylose reductase (XR) and xylitol dehydrogenase (XDH), as a result of which d-xylulose is produced. The resulting xylulose is then phosphorylated by xylulokinase (XK), and subsequently utilized by the endogenous pentose phosphate pathway followed by glycolysis to produce ethanol [7–11]. However, because of the differences in the cofactor preferences of NADPH-dependent XR and NAD^+^-dependent XDH, substantial amount of xylitol is accumulated, and thus, only suboptimal yield of ethanol is obtained when a recombinant yeast harboring the XR-XDH pathway is used for this purpose.

The second pathway consists of one step conversion of xylose to fermentable xylulose by xylose isomerase (XI) without the involvement of redox cofactors. A large number of XIs have been heterologously expressed in *S. cerevisiae*, and several of them were found to be functional [2, 12–27]. A XI gene that expressed highly functional XI activity in *S. cerevisiae* was first cloned from the anaerobic fungus *Piromyces* sp. strain E2, isolated from the feces of an Indian elephant [13, 28]. The *Piromyces* XI gene was overexpressed in a *S. cerevisiae* strain [29], and after long-term adaptation to xylose and following extensive genetic engineering of the strain [30], the recombinant strain yielded high amount of ethanol from xylose without any accumulation of xylitol. Thereafter, two XI genes, one from the cattle rumen fungus *Orpinomyces* sp. strain *ukk1* and another from the anaerobic bacterium *Clostridium phytofermentans*, were functionally expressed in *S. cerevisiae*. The *Orpinomyces* XI was highly homologous to the *Piromyces* XI (94% amino acid sequence identity), and both exhibited similar specific enzyme activities [14]. The *Clostridium* XI, on the other hand, exhibited lower sequence identity (<60%) to the *Piromyces* XI. The *Clostridium* XI was reported to be less inhibited by xylitol compared to the *Piromyces* XI [12].

Recently, two other XI genes, isolated from a soil metagenomic library, were shown to be functionally active in *S. cerevisiae* [18]. Although the enzyme activities of these XIs, expressed in *S. cerevisiae*, were comparable to that of the *Piromyces* XI, the aerobic growth rate of the recombinant yeast strain, expressing either one of these XI genes, on xylose was much lower than the growth rate of the yeast strain expressing the *Piromyces* XI. More recently, a new XI gene isolated from the bovine rumen metagenomics library was functionally expressed in *S. cerevisiae* and the expressed XI activity was improved through mutagenesis [27].

In this paper, we first report the cloning of a novel XI gene (cDNA) from the protists residing in the termite hindgut and then show that the xylose fermentation capacity of the recombinant *S. cerevisiae* strain expressing this clone is superior to previously known XI clones. Analysis of the kinetic properties of the protist XI reveals that the substrate specificity of this XI for xylose is higher than that of the other known XIs, suggesting that the expressed XI may rapidly consume low concentration of xylose from the fermentation medium. In addition, we discovered that a point mutation in the XI gene leads to improved cell growth on xylose and also leads to an increased xylose consumption rate for the yeast expressing this mutant XI. Furthermore, we show that introduction of the same mutation in XIs from various other organisms also improves their respective performances.

## Results

### Isolation of novel XI genes and their sequence analysis

Novel XI genes were isolated from a cDNA library, which was constructed previously from the protists residing in the *Reticulitermes speratus* hindgut [31], using a PCR-based cloning method. Briefly, degenerate primers, designed based on the alignment of DNA sequences of known XI genes, were used to amplify fragments with XI gene-like partial sequences. Next, the 5′- and 3′-regions of these XI gene-like fragments were amplified by PCR from the same cDNA library using primers that were designed based on the internal sequences of these XI gene-like fragments and the vector sequences flanking the 5′- and 3′-regions of the cDNA inserts. Finally, the entire open reading frame containing DNA fragment of each clone was amplified from the cDNA library using primers that were designed based on the DNA sequences of 5′- and 3′-regions of the XI gene-like fragments. DNA sequencing analysis of these clones led to the identification of eight novel XI genes and they were named as *RsXI*-*A* (GenBank Accession Number: LC272084), -*B* (GenBank Accession Number: LC272085), -*C1* (GenBank Accession Number: HV438106.1), -*C2* (GenBank Accession Number: HV438107.1), -*D1* (GenBank Accession Number: HV438108.1), -*D2* (GenBank Accession Number: LC272086), -*E* (GenBank Accession Number: LC272087), and -*F* (GenBank Accession Number: HV438109.1).

The amino acid sequences of eight novel XI genes were derived from their respective DNA sequences, and sequence homologies among these proteins were compared. Based on their sequence identities, these genes were divided into two groups. Whereas RsXI-A and RsXI-B exhibited 68.9% amino acid identity between them, RsXI-C1, RsXI-C2, RsXI-D1, RsXI-D2, RsXI-E, and RsXI-F revealed 81–92% amino acid identity among themselves. However, the degree of identity between these two groups was low (52% or less).

**Fig. 1:**
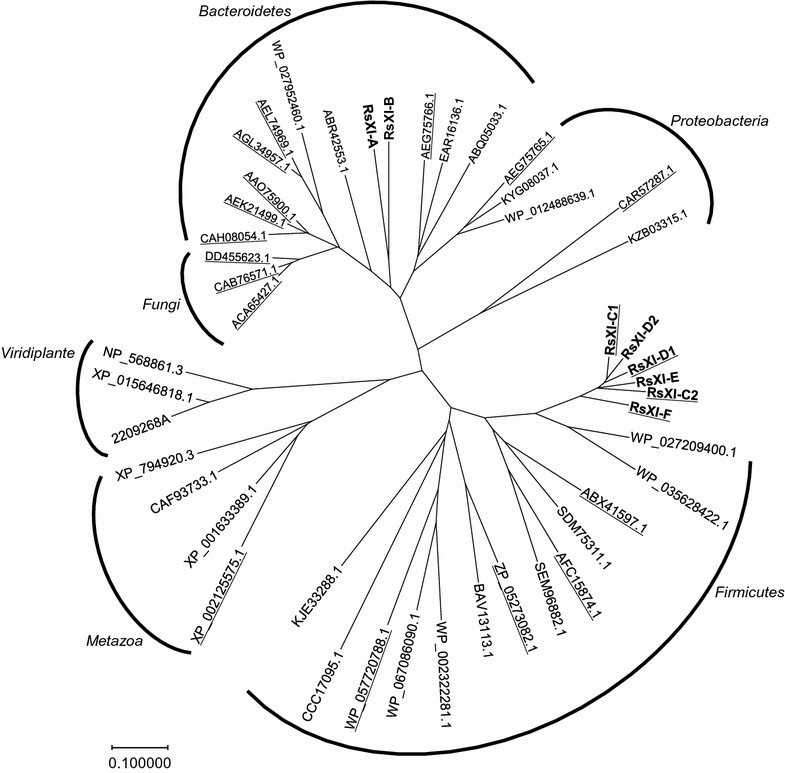
Phylogenetic tree. Phylogenetic analysis based on the amino acid sequences of the clones isolated in this study (*bold*) and other known XIs. Organism sources were selected based on their functional expression in *S. cerevisiae* (*underlined*) and sequence similarity. Protein names or protein accession numbers are given

Next, we performed phylogenetic analysis of these newly cloned XIs and XIs cloned from other sources, including those that are known to be functional in *S. cerevisiae*, such as XI genes of *Piromyces* sp. E2 and *C. phytofermentans*. As shown in Fig. 1, RsXI-A and RsXI-B showed relatively high similarities to the XIs from the *Bacteroidetes* family. The XIs from the *Fungi* family, including those from *Piromyces* sp. E2, were close to this phylum. However, RsXI-C1, RsXI-C2, RsXI-D1, RsXI-D2, RsXI-E, and RsXI-F were comparatively similar to the XIs from the *Firmicutes* family. These six novel XIs exhibited 63.4, 60.9, 62.6, 62.9, 60.1, and 62.2% identity, respectively, to the XI from *C. phytofermentans* (Additional file 1: Table S1). The results of phylogenetic analysis suggested that these XIs from the protists may belong to a phylogenetic affiliation that is different from the previously reported XIs.

### XI activities of novel XIs in recombinant yeasts

To determine whether these novel XI genes could be functionally expressed in the yeast *S. cerevisiae*, we assayed for xylose isomerase activity in cell extracts of *S. cerevisiae* harboring plasmids expressing these XI genes. The yeast strains used in this experiment are listed in Additional file 2: Table S2. In this assay, xylose is isomerized to xylulose by the xylose isomerase present in the crude cell extracts, and the amount of xylulose produced is determined by the cysteine-carbazole-sulfuric acid method [32].

**Fig. 2:**
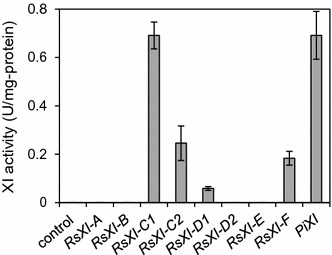
Xylose isomerase activities of the novel XI genes and *Piromyces* sp. E2 XI gene. Each gene was expressed in *S. cerevisiae* PP600 strain and xylose isomerase activity in the cell extract of each recombinant yeast strain was determined as described in "Methods". *Error bars* represent standard deviations of 4 independent experiments

As shown in Fig. 2, no XI activity was observed when the cell extract was prepared from the control strain carrying the empty vector. Similarly, XI activity was also not observed when the cell extract was prepared from the strain harboring the plasmid expressing *RsXI*-*A*, *RsXI*-*B*, *RsXI*-*D2*, or *RsXI*-*E*. XI activity was, however, detected in the cell extract prepared from the MP110 strain that harbored the XI gene from *Piromyces* sp. E2 (*PiXI*). More importantly, significant level of XI activity was also detected in the cell extracts prepared from the recombinant strain harboring the plasmid expressing *RsXI*-*C1*, *RsXI*-*C2*, *RsXI*-*D1,* or *RsXI*-*F*. Among these, the XI activity observed in the cell extract of MR310 strain, which expressed *RsXI*-*C1*, was equivalent to that of the MP110. Because the XI activity was highest in the yeast expressing *RsXI*-*C1*, we chose this clone for further characterization.

### Determination of the origin of *RsXI*-*C1*

Next, we determined the origin of *RsXI*-*C1*. Although it is likely that the origin of this gene is the symbiotic eukaryotic protists of *R. speratus* because the cDNA library, from which this gene was isolated, was constructed by the poly-T selection-based method, it is however possible to get contaminated by genes from the symbiotic bacteria. Therefore, we used RT-PCR and in situ hybridization techniques to determine the organism from which this gene was originated.

For this purpose, we first fractionated symbiotic protists and bacteria present in the termite hindgut by low-speed centrifugation and then performed RT-PCR on each fraction to determine which fraction would contain *RsXI*-*C1*. Accordingly, *RsXI*-*C1* was detected only in the protistan fraction (Additional file 3: Figure S1).

**Fig. 3:**
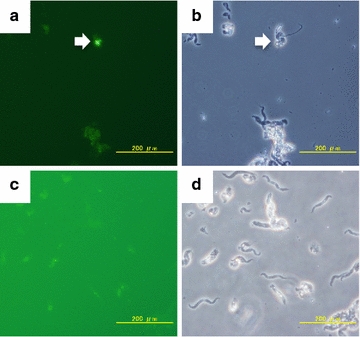
In situ hybridization. **a** Fluorescent image of in situ hybridization performed using the anti-sense probe. The picture was taken in 14-s exposure. Hybridization signal was indicated by the *arrowhead*. **b** Optical image of the same specimen shown in *panel* **a**. *Arrowhead* shows the individual that exhibited positive signal in *panel* **a**. **c** Fluorescent image of in situ hybridization performed using the sense probe. The picture was taken in 60-s exposure. No hybridization signal was observed. **d** Optical image of the same specimen shown in *panel* **c**. *Bar* 200 μm

To further confirm that *RsXI*-*C1* is only expressed in protists, we performed in situ hybridization analysis. The hybridization signal was observed only with the anti-sense probe (Fig. 3a, b), but not with the sense probe (Fig. 3c, d), in small number of protists, morphologically which were thought to be of the genus *Pyrsonympha* from their appearances (spiral cell and asymmetric shape along anterior–posterior axis [33]). This result suggests that either the *RsXI*-*C1* expressing protists are quite rare or they express *RsXI*-*C1* only under specific condition.

### Kinetic properties of RsXI-C1

For the kinetic studies, we used two recombinant *S. cerevisiae* strains, WR311 and WP111, expressing codon-optimized RsXI-C1 and PiXI, respectively. Codon optimization was carried out to improve the expression levels of these two genes in *S. cerevisiae*. Kinetic properties of RsXI-C1 and PiXI were determined using the crude cell extracts of these strains and various xylose concentrations. The kinetic parameters were calculated from the Lineweaver–Burk plot (xylose isomerase activity vs. xylose concentration). The *V*_max_ of PiXI (0.0103 μmol/min mg/protein) was about 1.4 times higher than that of the RsXI-C1 (0.0074 μmol/min mg/protein), but the *K*_m_ value of RsXI-C1 for xylose was 30% lower than that of the PiXI, thus suggesting that the affinity of the RsXI-C1 for xylose (10.52 ± 1.10 mM) was higher than that of PiXI (14.90 ± 0.57 mM).

### Growth of the yeast strain expressing *RsXI*-*C1O* on xylose

**Fig. 4:**
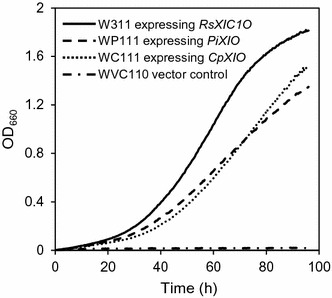
Aerobic growth of recombinant *S. cerevisiae* strains expressing different XIs on xylose. Yeast strains were cultivated aerobically at 30 °C in L-shaped test tubes in the xylose minimal medium containing 50 g/l xylose as the sole carbon source (see "Methods"). The data points shown are averages of three independent experiments. Deviations were always below 10% of the average

To evaluate the growth performance, growths of strains WR311 (RsXI-C1), WP111 (PiXI), and WC111 (expressing codon-optimized XI gene from *C. phytofermentans*, CpXI) were monitored in a medium containing xylose as the sole carbon source (SX medium). The results of the growth assay are shown in Fig. 4. As can be seen, OD_660_ of the control cell culture (WVC110 strain harboring control vector) did not increase, but the OD_660_ of the cultures of all the strains harboring the XI genes increased with culture time. The yeast strain WR311 (RsXI-C1) grew faster than the strains WP111 (PiXI) and WC111 (CpXI). The maximal specific growth rate of the WR311 strain (0.071 h^−1^) was about 1.2 times higher than the specific growth rates of the WP111 and WC111 strains (0.058 and 0.057 h^−1^, respectively).

### Fermentation ability of the yeast strain harboring the *RsXI*-*C1O* expression plasmid

Xylose fermentation abilities of the recombinant yeast strains were estimated from the amount of xylose consumed and ethanol produced in a medium that either contained 50 g/l xylose as the sole carbon source or contained a mixture of 30 g/l glucose and 20 g/l xylose.

**Fig. 5:**
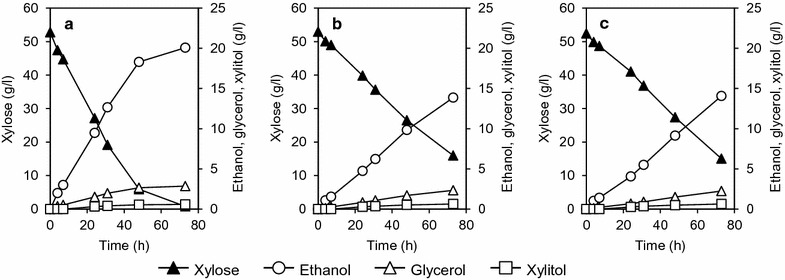
Anaerobic batch fermentation on xylose. Fermentation of xylose by WR311 (**a**), WP111 (**b**), and WC111 (**c**) strains under anaerobic condition was carried out in SX medium containing 50 g/l xylose as the sole carbon source (see "Methods"). Amounts of fermentation products (ethanol, glycerol, and xylitol) are indicated on the right-hand *Y-axis*. The data points shown are averages of three independent experiments. Deviations were always below 10% of the average

In the xylose fermentation assay, the initial xylose consumption rate of WR311 was twice as high as the xylose consumption rates of WP111 and WC111 (Fig. 5). After 72-h cultivation, xylose in the culture medium of WR311 was depleted, whereas 15 g/l or more xylose remained in the culture media of WP111 and WC111. The xylose consumption rates of WR311, WP111, and WC111 were 0.71, 0.51, and 0.51 g xylose/l h, respectively. In all cases, accumulation of only a little amount of xylitol (0.64 g/l or less) and some glycerol (2.9 g/l or less) was observed after 72-h fermentation. As a result, the ethanol yields of WR311, WP111, and WC111 were 0.39, 0.38, and 0.38 g of ethanol produced per g of consumed xylose, respectively.

**Fig. 6:**
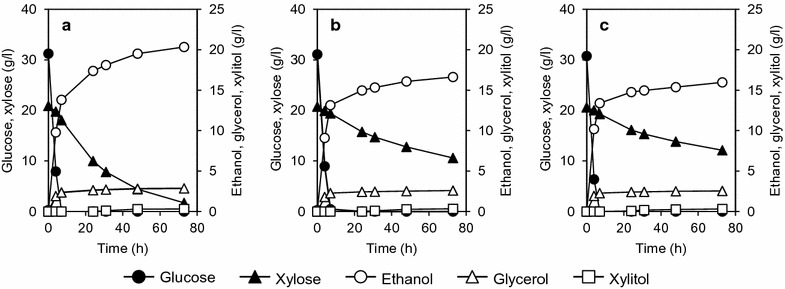
Anaerobic batch fermentation on glucose/xylose mixture. Fermentation of glucose/xylose by yeast strains WR311 (**a**), WP111 (**b**), and WC111 (**c**) was carried out in medium containing 30 g/l glucose and 20 g/l xylose under anaerobic condition. Amounts of ethanol, glycerol, and xylitol produced as a result of fermentation are indicated on the right-hand *Y-axis*. The data points shown are averages of three independent experiments. Deviations were always below 10% of the average

The time course of glucose/xylose fermentation by the recombinant yeast strains is shown in Fig. 6. As shown, glucose was preferably consumed at the initial stage of fermentation by all transformants, and xylose concentration only began to decrease as the glucose was depleted from the culture medium. In all cases, we found almost no accumulation of the by-product xylitol. WP111 and WC111 consumed less xylose; thus, 10 g/l or more xylose remained in the culture media of WP111 (Fig. 6b) and WC111 (Fig. 6c) after 72-h fermentation. On the other hand, the xylose consumption rate of the WR311 strain was about twice that of the other strains, and almost all the xylose was consumed after 72-h fermentation (Fig. 6a).

To ensure that the higher xylose fermentation ability of WR311 was indeed due to the expression of the XI gene and was not influenced by the host strain, we used another host strain, PP600, and created recombinant strains expressing codon-optimized *RsXI*-*C1*, *PiXI,* and *CpXI*. We then repeated the fermentation experiment using these recombinant PP600 strains under microaerobic condition under xylose minimal medium and the results are shown in Additional file 4: Figure S2. As was observed with the recombinant W600W strains, the recombinant PP600 strain expressing *RsXI*-*C1* exhibited higher xylose consumption rate than the other recombinant strains, thus suggesting that the observed higher xylose isomerase activity of RsXI-C1 was not host strain dependent.

It is also believed that the background status of host strain, such as the expression levels of genes related to PPP or sugar transport, might influence the xylose fermenting ability. To test this possibility, we analyzed the expression levels of these genes in each recombinant W600W strain (i.e., WR311, WP111, and WC111) as well as in control strain by quantitative PCR. Results shown in Additional file 5: Figure S3 revealed that although there were some differences in the expression levels of these genes, the observed differences were not significant, indicating that the background strains are at the same status. Collectively, these results suggest that the RsXI-C1 expressed in yeast can utilize xylose more efficiently than that either by PiXI or by CpXI.

### Screening and identification of xylose isomerase mutants with improved growth on xylose

We performed random mutagenesis of *RsXI*-*C1O* followed by growth-based screening on xylose to select for a mutant XI gene that would confer enhanced ability to ferment xylose in the recombinant *S. cerevisiae* strain. Mutated DNA fragments and the native *RsXI*-*C1O* were transformed separately into the W600W strain using a low-copy-number centromeric vector pRS316GAP. For growth-based screening, yeast transformants, harboring a plasmid expressing either the wild-type or a mutant *RsXI*-*C1O*, were cultured in the SX medium. After long-term cultivation, cells were harvested and spread on a SX agar plate. Next, 20 colonies, growing faster than the control strain and harboring mutant *RsXI*-*C1O*, were selected from the SX agar plate for the growth experiment in SX medium (Additional file 6: Figure S4). Based on the specific growth rates of these 20 colonies, top 10 colonies with better growth rates were selected and used for plasmid preparation.

**Fig. 7:**
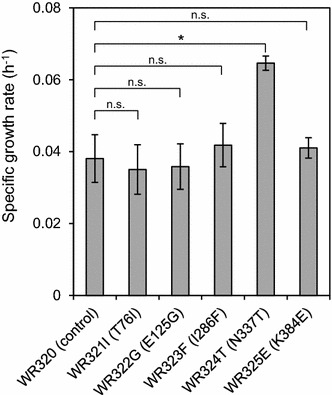
Specific growth rates of the yeast strains expressing mutated RsXI-C1s. Growth of cells, harboring either the wild-type RsXI-C1 (control) or point mutants of RsXI-C1 (T76I, E125G, I286F, N337T, or K384E), was carried out in SX medium under aerobic condition. Growth rates were determined as described in "Methods". *Error bars* indicate standard deviations of biological quintuplicates. Statistical significance was determined using Student’s *t* test. *P < 0.05, significant difference. *n.s.* no significant difference

Results of the sequencing analysis revealed 5 different mutant sequences. Notably, each mutant XI gene contained a single amino acid substitution at one of the following amino acid residues of RsXI-C1: T76I, E125G, I286F, N337T, and K384E. Genes corresponding to these mutants were designated as *RsXIC1O*-*T76I*, *RsXIC1O*-*E125G*, *RsXIC1O*-*I286F*, *RsXIC1O*-*N337T*, and *RsXIC1O*-*K384E*, respectively. Plasmids carrying each one of these mutated XI genes were transformed into the W600W strain, and the resulting strains are summarized in Additional file 2: Table S2. A growth assay was performed to evaluate the growths of these recombinant yeasts on xylose. The specific growth rates of these mutants are compared in Fig. 7. Among these, only the specific growth rate of the WR324T (RsXIC1O-N337T) strain was 1.6-fold higher than that of the control strain.

### Fermentation performance of the N337T XI mutant expressing strain

**Fig. 8:**
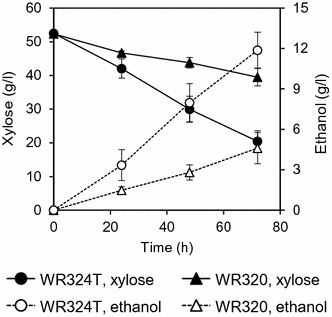
Time course of anaerobic batch fermentation on xylose. *S. cerevisiae* strains WR324T (expressing *RsXIC1O*-*N337T* mutant) and WR320 (expressing wild-type *RsXI*-*C1O*) were cultivated in the fermentation medium that contained 50 g/l xylose as the sole carbon source under anaerobic condition. Concentrations of xylose remaining in the medium (*closed symbols*) and ethanol produced during the fermentation (*open symbols*) at the indicated times were determined by HPLC. *Error bars* represent standard deviations of four independent experiments

As mentioned above, the yeast strain expressing the N337T mutant of RsXI-C1 (WR324T) exhibited enhanced growth rate on xylose. Therefore, to evaluate the xylose fermentation ability of the WR324T (N337T) strain, xylose fermentation experiments were carried out under anaerobic condition in 50 ml of SX medium containing 50 g/l xylose (initial OD_600_ = 10). Figure 8 shows time-dependent changes in xylose and ethanol concentrations in the fermentation medium of the strains WR324T and WR320 (control strain). As shown, the amount of xylose consumed by WR320 in 72 h was 12.9 g/l and that by WR324T during the same period was 32.0 g/l. Thus, the xylose consumption and the ethanol production rates for the WR324T strain were 2.5 times higher than those for the WR320 strain.

### Construction and evaluation of saturated mutagenesis library

**Fig. 9:**
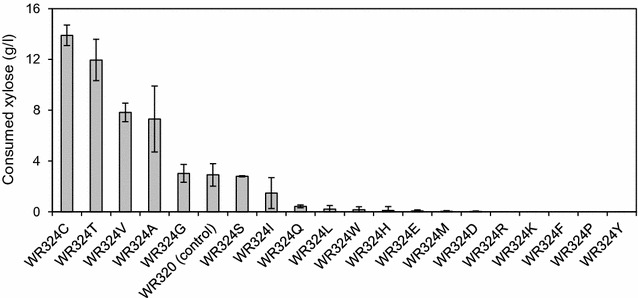
Xylose fermentation by recombinant yeast strains expressing single amino acid substitution mutants of RsXI-C1. Xylose consumed by *S. cerevisiae* cells expressing the indicated RsXI-C1 point mutants, obtained by site-saturation mutagenesis of Asn-337, after 72-h fermentation under microaerobic condition. Xylose fermentation was carried out as described in "Methods". In all cases, initial cell density (absorbance at 600 nm, OD_600_) was 1.0. *Error bars* indicate standard deviations of biological duplicates or triplicates

Next, we examined whether replacement of the N337 residue of RsXI-C1 with other amino acids would improve the xylose utilization ability. For this purpose, a site-saturation mutagenesis library was constructed by site-directed mutagenesis using pRS316GAP-RsXIC1O as the template. The resulting plasmids, each carrying a mutant *RsXI*-*C1O* in which the N337 residues was substituted with one of the remaining amino acid residues, were separately introduced into the W600W strain to generate eighteen mutant strains (Additional file 2: Table S2). These mutant strains were then tested for their abilities to ferment xylose using the microaerobic fermentation assay. Figure 9 shows xylose consumption by these mutant and control yeast strains after 72-h incubation. Xylose consumed by the WR320 strain (control) at 72 h was 2.9 g/l, and that by the WR324T (N337T) strain at 72 h was 12.0 g/l. Out of 18 different single amino acid substitution mutants, WR324C (N337C), WR324V (N337V), and WR324A (N337A) consumed more xylose (13.9, 7.8, and 7.3 g/l, respectively) than WR320. Among these mutants, the WR324C strain consumed the highest amount of xylose (4.8-fold more than WR320). From these results, it became clear that the xylose utilization ability of yeast could be improved by substituting the asparagine at position 337 of RsXI with threonine, cysteine, valine, or alanine.

### Effect of mutation on the xylose utilization abilities of other XIs

We next examined whether introduction of the same point mutation would similarly improve the xylose utilization abilities of other XIs, such as PiXI, CpXI, and the XI from *Lactococcus lactis* (LlXI) [15], all of which were functionally expressed in *S. cerevisiae*.

**Fig. 10:**
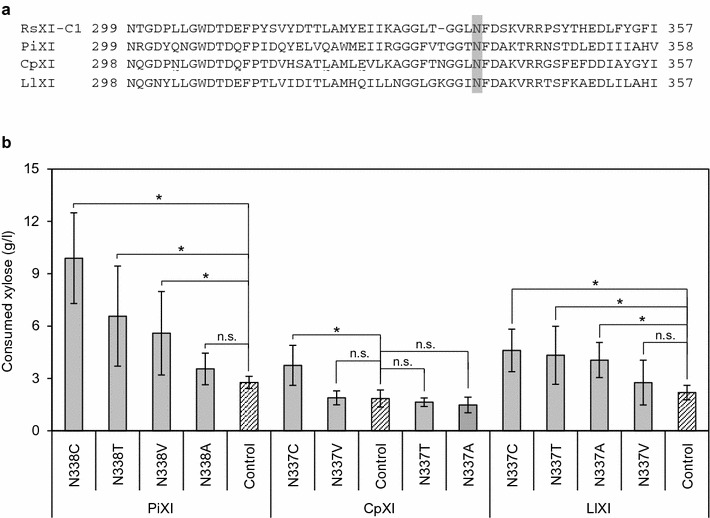
**a** Alignment of amino acid sequences of different XIs. RsXI-C1, novel XI cloned from the cDNA library of symbiotic protists of *R. speratus*; PiXI, XI of *Piromyces* sp. E2; CpXI, XI of *C. phytofermentans*; LlXI, XI of *L. lactis*. Amino acid sequence alignment was carried out using the genetic information analysis software GENETIX version 10. The *shaded column* indicates the point mutation site. **b** Xylose fermentation by recombinant yeast strains expressing mutated PiXI, CpXI, and LlXI. Xylose consumed after 72-h fermentation under microaerobic condition was shown. Xylose fermentation was carried out as described in "Methods". In all cases, initial cell density (absorbance at 600 nm, OD_600_) was 10.0. *Error bars* represent standard deviations of biological tetraplicates. Statistical significance was determined using Student’s *t* test. *P < 0.05, significant difference. *n.s.* no significant difference

Alignment of the amino acid sequences of RsXI-C1, PiXI, CpXI, and LlXI (Fig. 10a) revealed that the amino acid residue corresponding to the asparagine 337 of RsXI-C1 was also an asparagine residue in all of them; interestingly, amino acid sequences adjacent to this asparagine were found to be highly conserved. Therefore, we substituted the corresponding asparagine residue in these three XIs with an alanine, a cysteine, a threonine, or a valine residue. The generated mutant genes and each native wild-type XI gene were transformed into the W600W strain using the low-copy vector pRS316GAP. The resulting plasmids are listed in Additional file 7: Table S3, and the resulting recombinant yeasts are listed in Additional file 2: Table S2.

Xylose utilization abilities of yeast strains harboring these mutant plasmids were assessed as before. Figure 10b shows the amount of xylose consumed by each one of these recombinant yeast strains after 72-h incubation. Xylose consumed by WP120 (expressing wild-type PiXI) after 72-h fermentation was 2.8 g/l; in contrast, xylose consumed by the PiXI mutant strains WP124C (N338C), WP124T (N338T), and WP124V (N338V) were 9.9, 6.6, and 5.6 g/l, respectively, indicating their improved xylose utilization ability. Similarly, strains harboring cysteine substitution mutant of CpXI (N337C), and cysteine, threonine, or alanine substitution mutant of LlXI (N337C, N337T, or N337A) also exhibited improved xylose utilization ability.

Taken together, our results suggest that the N337 of RsXI-C1 as well as the corresponding asparagine residues of PiXI, CpXI, and LlXI might play an important role in recognizing xylose, because mutagenesis of this residue conferred improved xylose utilization in yeast.

## Discussion

Yeast *S. cerevisiae* has been used for industrial bioethanol production because of its superior capacity, such as robust fermentation, inhibitor tolerance, and high ethanol productivity [1, 34]. The XI pathway, which can circumvent the redox cofactor imbalance, has been proposed as the most efficient pathway in *S. cerevisiae* to produce ethanol from xylose [29]. Several XI genes have been reported to be functional in *S. cerevisiae*. Thus, the recombinant yeast strains expressing the XI genes isolated from *Piromyces* sp. E2, *C*. *phytofermentans*, *Orpinomyces*, and *Bacteroides* fermented xylose efficiently after long-term adaptation to xylose and extensive genetic engineering [2, 14, 26, 35–38]. Here, for the first time, we report isolation of novel XI clones from the protists residing in the termite hindgut; these newly cloned XI genes exhibited low sequence identity with other known XI genes, several of which were functionally expressed in *S. cerevisiae*. Although we performed multiple homology searches using the amino acid sequences of the newly found XIs, we failed to identify any distinctive feature that would explain why some of the XIs are active while the others are inactive when expressed in *S. cerevisiae*. The amino acid sequence derived from *RsXI*-*C1*, one of the newly isolated XI genes that exhibited the highest XI activity when expressed in yeast (Fig. 2), shared about 47–63% sequence identity with the amino acid sequences of other functional XIs (Additional file 1: Table S1). Although these novel XIs from protists were comparatively similar to the XIs from *Firmicutes,* they formed a slightly different cluster in the phylogenetic tree analysis (Fig. 1). This may be the result of evolutionary changes in the XI genes under a unique environment, such as the termite gut.

Our RT-PCR assay and in situ hybridization results showed that the *RsXI*-*C1* expressing protists might be of genus *Pyrsonympha*. However, in situ hybridization signal was not observed in most *Pyrsonympha*—signals were observed only from a few small size individuals. It is known that *Pyrsonympha* have several morphologically uncharacterized taxonomy units [39]; *Pyrsonympha* that expressed *RsXI*-*C1* might be one of such morphologically uncharacterized species.

The kinetic analyses revealed that the *V*_max_ value of RsXI-C1 was 30% lower than that of PiXI, and the *K*_m_ value of RsXI-C1 for xylose (10.52 ± 1.10 mM) was 30% lower than that of PiXI (14.90 ± 0.57 mM). The lower *K*_m_ value of RsXI-C1 suggests that the affinity for xylose was higher for RsXI-C1 than that for PiXI. As *S. cerevisiae* is known not to have any specific xylose transporter, this yeast is believed to take up xylose into the cell via the non-specific glucose transporters. Considering that the glucose transporters exhibit a significantly lower affinity for xylose (*K*_m_ = 49–300 mM) than for glucose (*K*_m_ = 1–28 mM) [7], intracellular xylose concentration would be low, especially when the xylose concentration becomes lower as the xylose gets consumed. Because of the high affinity of XI for xylose, the yeast could convert xylose to xylulose more efficiently at such low xylose concentration. Thus, rapid consumption of xylose would be expected in yeast expressing the higher affinity XI. Indeed, the recombinant yeast strain expressing RsXI-C1 consumed xylose with higher rate under anaerobic batch condition in xylose minimal medium (Fig. 5) as well as in mixed glucose and xylose medium (Fig. 6), and also under microaerobic fermentation condition (Additional file 8: Figure S5) than the strain expressing either PiXI or CpXI. Since only small amount of xylitol was produced, the yield of ethanol was quite high (0.39 g/g in xylose medium and 0.40 g/g in mixed glucose and xylose medium). Clearly, enhanced ability to ferment xylose led to higher ethanol titer and shorter fermentation time that can greatly reduce the production cost of bioethanol. It is noteworthy that expression of *RsXI*-*C1O* in the yeast strain W600W, in which XK and non-oxidative pentose phosphate pathway genes have been overexpressed and endogenous aldose reductase gene *GRE3* has been deleted, led to marked increase in cell growth and fermentation efficiency in xylose minimal medium without the evolutionary adaptation process.

So far, many attempts have been made to improve the activity of recombinantly expressed xylose isomerase in *S. cerevisiae* [15, 19, 27, 40–42]. To obtain a RsXI-C1 that would confer enhanced xylose fermentation in recombinant yeast, we carried out random mutagenesis of *RsXI*-*C1O* coupled with growth-based screening on xylose. Consequently, we isolated the N337T mutant of RsXI-C1, which when expressed in yeast exhibited 1.6-fold higher specific growth rate on xylose (Fig. 7) and consumed 2.5 times more xylose (Fig. 8) than the yeast strain expressing the wild-type RsXI-C1. Moreover, analysis of substitution mutants of the asparagine 337 residue of RsXI-C1 revealed that substitution of the asparagine 337 to cysteine further improved the xylose utilization ability of the recombinant yeast (Fig. 9). In addition, substitution of the corresponding asparagine residues of PiXI, CpXI, and LlXI with alanine, cysteine, threonine, or valine improved their abilities to utilize xylose (Fig. 10).

Previous reports showed that introduction of mutations in the XIs from *Piromyces* sp. E2 and bovine rumen metagenomic library improved their enzyme activities [27, 40]. It is thought that these mutations might increase the activity of XI by increasing the monomer-binding contacts or substrate enzyme interactions. Additional file 9: Figure S6 shows the predicted three-dimensional models of the active sites of RsXI-C1 and N337C mutant of RsXI-C1. Although the residue Asn337, which was subjected to mutagenesis, is distantly located from the active site residues (Phe101, His102, Asp103, and Lys235), our modeling result suggested that this residue is sitting close to Asp339 at the metal binding site. The side chain of Asn337 is also close to the metal biding site. It seems that the change occurred in the metal binding site, as a result of replacement of the asparagine residue by the cysteine residue, may have contributed to improved XI performance.

These findings demonstrate the importance of the N337 residue in improving the xylose consumption capacity of RsXI-C1, as XIs from several other organisms, all of which were functionally expressed in *S. cerevisiae*, also shared the same characteristics.

In this study, we reported cloning of novel XI genes from the protists residing in the termite hindgut and successfully expressed some of these clones in *S. cerevisiae*. One of these XI genes, named here as *RsXI*-*C1*, expressed high XI activity in yeast, and the expressed RsXI-C1 exhibited higher substrate affinity for xylose than those reported earlier for other XI genes expressed in yeast. The recombinant strain expressing RsXI-C1 fermented xylose efficiently. To further improve xylose fermentation, it might be necessary to carry out strain engineering including strong and stable expression of *RsXI*-*C1* from a multicopy genomic integration vector targeted to the rDNA cluster, introduction of an efficient sugar transporter, tuning of the genes related to xylose metabolism, use of industrial diploid yeast strains, and perform long-term adaptive evolution on xylose [5, 6, 37, 38], individually or in combination.

## Conclusions

In this study, novel XI coding genes were successfully isolated from a cDNA library of protists residing in the *R*. *speratus* hindgut. The recombinant *S. cerevisiae* strain expressing *RsXI*-*C1* exhibited better growth rate and xylose fermentation ability than those strains expressing other known XI genes, including the XI genes from *Piromyces* and *Clostridium*. Furthermore, a single amino acid substitution mutant of RsXI-C1, obtained by random mutagenesis of *RsXI*-*C1* combined with growth-based screening on xylose, improved xylose fermentation by the yeast strain expressing this mutant. In addition, mutants carrying similar single amino acid substitutions at the corresponding asparagine residues of several other XIs, previously cloned from different organisms, also conferred improved ability to ferment xylose in *S. cerevisiae*.

## Methods

### Strain and media

The *Escherichia coli* DH5∝ (Nippon gene, Japan) was used for all recombinant DNA manipulations. All the yeast strains used and constructed in this study are summarized in Additional file 2: Table S2. *S. cerevisiae* strains MT8-1 [43] and W303-1B [44] were used as the host strains for the gene expression and fermentation, respectively. *E. coli* was grown in Luria–Bertani medium (10 g/l tryptone, 5 g/l yeast extract, 5 g/l sodium chloride) containing 100 mg/l ampicillin or 50 mg/l kanamycin. *S. cerevisiae* strains were aerobically cultivated at 30 °C in synthetic medium [SD medium; 20 g/l glucose and 6.7 g/l yeast nitrogen base without amino acids (Difco/BD Diagnostic Systems, MD, USA) with appropriate supplements.

Adaptation to xylose is known to greatly improve the xylose fermentation abilities of the recombinant yeast strains [29, 35]. Thus, to exclude the effect of adaptation, we used glucose as the carbon source to prepare the pre-cultures used for the enzyme and growth assays, and also in fermentation experiments.

### PCR amplification and cloning procedure

PCR reactions were carried out either with PrimeSTAR HS DNA polymerase or with Ex Taq HS DNA polymerase (Takara Bio Inc., Japan). The PCR primers used in this study are listed in Additional file 10: Table S4. PCR products were cloned into pCR2.1-TOPO using the TOPO TA cloning kit or pCR-BluntII-TOPO using the ZERO Blunt TOPO PCR cloning kit (Thermo Fisher Scientific Inc., MA, USA). For generating plasmids, the DNA fragments were cloned into appropriate vectors using the In-Fusion Advantage PCR Cloning kit (Takara Bio Inc.).

### Isolation of novel XI genes from cDNA library

A cDNA library, previously prepared from the protists residing in the *R. speratus* hindgut [31], was used in this study for the cloning of novel XI genes. In order to obtain a xylose isomerase gene from this cDNA library, degenerate primers mXI-F1 and mXI-R1, which were designed based on the alignment of DNA sequences of known XI genes obtained from GenBank, were synthesized. Using these degenerate primers, an approximately 400-bp DNA fragment was amplified from this cDNA library. The purified fragment was cloned and sequenced. Consequently, various plasmids containing inserts exhibiting partial sequence similarities to known XI genes were obtained. The 5′- and 3′-regions of these sequences were amplified from the cDNA library by PCR using appropriate combinations of the following primer pairs: RsA-F, RsB-R, RsC1-R, RsC2-F, RsD-R, RsE-F, and RsF-R, which were designed from the revealed partial sequences of inserts, and Lib-F and Lib-R, which were designed from the vector sequence of the 5′- and 3′-flanking regions of the cDNA insert. Amplified DNA fragments were cloned and sequenced. Next, full-length DNA fragments were obtained by PCR using the cDNA library as the template and appropriate primer pairs (RsA-termR, RsB-termF, RsC1-termF, RsC2-termR, RsD-termF, RsE-termR, and RsF-termF, all of which were designed from the 5′- or 3′-regions of the gene sequence, and Lib-F and Lib-R). Amplified DNA fragments were cloned and sequenced. Finally, the entire cDNA sequence of each clone was amplified by PCR from the cDNA library using one of the following appropriate primer pairs: RsXI-A-IF-F/RsXI-A-IF-R, RsXI-B-IF-F/RsXI-B-IF-R, RsXI-C1-IF-F/RsXI-C1-IF-R, RsXI-C2-IF-F/RsXI-C2-IF-R, RsXI-D-IF-F/RsXI-D-IF-R, RsXI-E-IF-F/RsXI-E-IF-R, or RsXI-F-IF-F/RsXI-F-IF-R. Each amplified fragment was then cloned into the *Sac*II/*Xho*I site of pRS436GAP (DDBJ Accession Number: AB3048629), and complete nucleotide sequence of each amplified DNA fragment was obtained. A general map of the resulting plasmid, created as above by inserting a RsXI gene into pRS436GAP, is shown in Additional file 11: Figure S7a. All the generated plasmids are listed in Additional file 7: Table S3.

### Phylogenetic analysis

The amino acid sequences derived from the full-length cDNA clones and DNA sequences of previously reported XIs were aligned and the phylogenetic tree was plotted using the genetic information analysis software GENETYX version 10 (GENETYX, Japan). The phylogenetic tree was constructed with 1000 boot strap replicates using the neighbor-joining method [45].

### RT-PCR procedure

To determine the origin of *RsXI*-*C1*, we fractionated symbiotic protists and bacteria, and performed RT-PCR on cDNA prepared from each fraction.

Excised hindguts from 30 individuals of *R. speratus* were placed into a 1.5-ml microfuge tube along with 100 μl of solution U [46] and disrupted by plastic pestle on ice. The resulting suspension was then filtered through a 100-μm pore size nylon mesh to remove the gut debris. The filtrate was centrifuged at 500 rpm for 5 min using a swing bucket rotor. The supernatant was used as the bacterial fraction and the pellet was used as the protistan fraction. To clean up the bacterial fraction, the supernatant was collected after centrifugation at 500 rpm for 5 min, and this cycle was repeated 4 times. The resulting supernatant was subsequently centrifuged at 15,000 rpm for 1 min and the resulting bacterial pellet was saved and the supernatant was discarded. To clean up the protistan fraction, the protistan pellet (see above) was resuspended in 100 μl of solution U, centrifuged at 500 rpm for 5 min, and the supernatant was discarded after centrifugation. This cycle was repeated 4 times. All centrifugation steps were performed at 4 °C.

Total RNA was purified from each fraction by RNeasy mini kit (QIAGEN, Hilder, Germany). RNA was eluted from the spin column with 30 μl of RNase-free water included in the kit. Eluted RNA was used as a template for RT-PCR.

The purified total RNA was reverse transcribed with the degenerated complementary primer RsXI_C1_Cend_comp, which was designed based on the C-terminus amino acid sequence of RsXI-C1. The transcribed mixture was then used in a PCR amplification reaction using the degenerate primer RsXI_C1_Cend_comp and the degenerated primer RsXI_C1_Nend, which was designed based on the N-terminus amino acid sequence of RsXI-C1.

Reverse transcription reaction was performed at 50 °C for 10 min using 200 units of Superscript IV (Thermo Fisher Scientific Inc.) in a total volume of 20 μl that contained 2 pmol of each primer, 1× Superscript IV buffer, 0.5 mM dNTP, and 5 mM DTT, following which the reverse transcriptase was inactivated by incubating the reaction mixture at 80 °C for 10 min. After the reaction, the remaining RNA was removed by treatment with RNase H (2 units per reaction) at 37 °C for 30 min.

The reaction product, obtained as above, was used for the PCR amplification. PCR reaction was carried out in 50 μl total volume and each reaction mixture contained 1× ExTaq buffer (Takara Bio Inc.), 50 pmol of each primer, 0.2 mM dNTP, 5 units ExTaq (Takara Bio Inc.), and 5 μl of transcribe product. The PCR reaction cycle used for amplification was as follows: 94 °C for 30 s, 50 °C for 45 s, and 72 °C for 2 min (total 30 cycles). The obtained amplicon was analyzed by 1% agarose electrophoresis.

### *In situ* hybridization

*In situ* hybridization was performed as described previously [47] with some modification. Excised hindguts isolated from 10 termites were placed into a 1.5-ml microfuge tube along with 100 μl of solution U and disrupted by plastic pestle on ice. The resulting suspension was then filtered through a 100-μm pore size nylon mesh to remove the gut debris.

Symbiotic protists were collected from the resulting suspension by centrifugation at 500 rpm for 3 min at 4 °C and the pellet was washed 3-times with 100 μl of solution U. Washed protists were fixed by incubating with solution U containing 4% formaldehyde at room temperature for 15 min. After fixation, suspended protists were dehydrated by sequentially washing with 50% ethanol for 5 min, 80% ethanol for 5 min, and finally with 100% ethanol for 5 min. After dehydration, suspended protists were fixed on glass slides by spotting and drying.

Spotted specimen was treated with pre-hybridization solution (2 pmol/μl RsXI_C1_Nend, 300 mM NaCl, 30 mM sodium citrate, and 0.01% SDS) at 37 °C for 30 min. After the removal of pre-hybridization solution, hybridization was carried out in hybridization solution that contained 2 pmol/μl fluorescein-labeled C_nested_comp (anti-sense) probe or fluorescent-labeled C_nested (sense) probe, 300 mM NaCl, 30 mM sodium citrate, and 0.01% SDS, and slides were incubated with this solution at 37 °C for 1 h. 5′ end fluorescein-labeled oligonucleotide probes were synthesized by Eurofins genomics K.K. (Tokyo, Japan). Probe sequences are listed in Additional file 10: Table S4.

After hybridization, slides were washed twice with PBS-T (0.4% NaCl, 0.01% KCl, 0.145% Na_2_HPO_4_·12H_2_O, 0.01% KH_2_PO_4_, 0.2% Tween 20) at 37 °C for 15 min to remove excess probes. Slides were then sealed with Vector Shield (Vector Laboratories, CA, USA) and observed under a fluorescent microscope (Model IX70, Olympus, Japan).

### Construction of plasmids

Expression plasmids were constructed to overexpress the following genes of the non-oxidative pentose phosphate pathway: *XKS1* encoding xylulokinase, *RK11* encoding ribulose 5-phosphate isomerase, *RPE1* encoding ribulose 5-phosphate epimerase, *TKL1* encoding transketolase, and *TAL1* encoding transaldolase. The integration vector pXhisHph-HOR7p-ScXK (Additional file 11: Figure S7b), which is targeted to the *HIS3* loci in chromosome XV, was used for the construction of *XKS1* overexpression plasmid. The integration vector pXAd3H-HOR7p-ScTAL1-ScTKL1 (Additional file 11: Figure S7c), which is targeted to the upstream region of *ADH3* in chromosome XIII, was used for the construction of *TAL1* and *TKL1* overexpression plasmids. The integration vector pXGr3L-HOR7p-ScRPE1-ScRKI1 (Additional file 11: Figure S7d), which is targeted to the *GRE3* loci in chromosome VIII, was used for the construction of *RPE1* and *RKI1* overexpression plasmids. All the genes were expressed under the control of the *HOR7* promoter and the *CYC1* terminator.

A codon-optimized *RsXI*-*C1* (*RsXI*-*C1O*) was synthesized based on the amino acid sequence of RsXI-C1. The XI gene from the *Piromyces* sp. E2 (*PiXI*) was synthesized based on the DNA sequence acquired from the GenBank (GenBank Accession Number: AJ249909.1). The codon-optimized version of *PiXI* (*PiXIO*) and codon-optimized XI gene from *C*. *phytofermentans* DSM18823 (*CpXIO*) were synthesized based on the amino acid sequences acquired from the GenBank (GenBank Accession Numbers: CAB76571.1 and ABX41597.1, respectively). These synthesized DNA fragments, which were custom synthesized from GenScript (NJ, USA), were individually cloned into the *Sac*II/*Xho*I site of pRS436GAP, and the resulting plasmids were designated as pRS436GAP-PiXI, pRS436GAP-PiXIO, pRS436GAP-RsXIC1O, and pRS436GAP-CpXIO, respectively (Additional file 7: Table S3).

### Yeast transformation

Yeasts were transformed with a Frozen-EZ Yeast transformation II kit (Zymo Research, CA, USA). About 1 μg of episomal plasmid or about 5 μg of linearized integration vector was used for the transformation, followed by selection on selective SD agar plates. Resulting yeast strains are summarized in Additional file 2: Table S2.

### Strain construction

To create a strain that would overexpress PPP-related genes, plasmids pXhisHph-HOR7p-ScXK, pXAd3H-HOR7p-ScTAL1-ScTKL1, and pXGr3L-HOR7p-ScRPE1-ScRKI1, each digested with restriction enzyme Sse8387I, were introduced into the strain MT8-1 and the resulting strain was named PP600.

W303-1B-based PPP overexpression strain was constructed as follows. First, the *ADE2* (GenBank Accession Number: M59824) was amplified by PCR from the genomic DNA purified from *S. cerevisiae* S288C and using the primer pair ADE2 + 1F and ADE2 + 1716R. The resulting amplification product was used to transform the W303-1B strain that complemented the *ade2* mutation. The resulting strain, which was named W303-1BA, was transformed with linearized DNA fragments obtained from the Sse8387I digested plasmids pXhisHph-HOR7p-ScXK, pXAd3H-HOR7p-ScTAL1-ScTKL1, and pXGr3L-HOR7p-ScRPE1-ScRKI1. The resulting strain was named W600.

Finally, the *TRP1* (GenBank Accession Number: 851570) and its neighboring region were amplified by PCR from the genomic DNA purified from *S. cerevisiae* S288C and using the primer pair TRP1M-F and TRP1M-R. The resulting amplification product was used to transform W600 strain to complement the *trp1* mutation, and the resulting strain was named W600W. Strains PP600 and W600W were transformed with the XI gene expression plasmids. Descriptions of the strains created in this study are listed in Additional file 2: Table S2.

### Enzyme assays

Yeast transformants were cultivated at 30 °C for 24 h in SD medium, following which cells were harvested by centrifugation, washed twice with sterile distilled water, and then washed twice with 100 mM phosphate buffer (pH 7.0). Washed cell pellets were disrupted with glass beads (diameter, 0.3 mm, Yasui Kikai, Japan) using Micro mixer E-36 (Taitec Corporation, Japan) or Multi-beads shocker (Yasui Kikai, Japan). Cell extracts were centrifuged at 12,000 rpm at 4 °C for 5 min, and the resulting supernatants were collected as crude cell extracts. The total protein concentration in the crude cell extract was determined using the Quick Start protein assay kit (Bio-Rad, CA, USA).

XI activities in the crude cell extracts of recombinant yeast strains were determined by two different assay methods. One method, which has been modified from a previously described method [32], was used for measuring the XI activity of novel XI gene products. In brief, the assay was carried out in a reaction mixture that contained 50 mM maleic acid (pH 6.85), 10 mM MgSO_4_, 1 mM CoCl_2_, 1 mM MnCl_2_, and 10 mM xylose. The reaction was initiated by the addition of crude cell extract, and then the reaction mixture was incubated at 30 °C for 30 min. After the incubation period was over, cysteine-carbazole-sulfuric acid regent, containing 2.7 mM cysteine, 0.22 mM carbazole, and 66% H_2_SO_4_, was added to the reaction mixture. The mixture was incubated for 20 min at room temperature, following which its absorbance at 540 nm was measured to determine the amount of d-xylulose produced using a d-xylulose standard curve. One unit (U) of enzyme activity was defined as the activity that produced one μmol d-xylulose per min at 30 °C.

The other method was used to determine the kinetic properties of XIs as described previously [12]. In this method, the XI activity was determined spectrophotometrically by measuring the oxidation of NADH at 340 nm. The assay mixture contained 100 mM Tris–HCl (pH 7.5), 10 mM MgSO_4_, 0.15 mM NADH, 2 U sorbitol dehydrogenase (Wako, Japan), and crude cell extract. The reaction was performed at 30 °C, and it was initiated by the addition of d-xylose to a final concentration of 5–250 mM.

### Growth assays

Yeast strains were grown on SD medium at 30 °C for 24 h. After cultivation, cells were collected, washed with sterile distilled water, and then inoculated into SX medium that contained 6.7 g/l of yeast nitrogen base without amino acids and 20 g/l of xylose as the sole carbon source. The initial cell density was adjusted to an optical density at 600 nm (OD_600_) of 0.05. For the growth assay, cells were grown in L-shaped test tubes at 30 °C with shaking (70 rpm) in a Bio-photorecorder (model TVS062CA, ADVANTEC, Japan).

### Anaerobic batch fermentation

Anaerobic batch fermentations were carried out in 100-ml medium bottles sealed with caps equipped with gas check valves. Yeast strains were aerobically pre-cultivated in SD medium for 3 days at 30 °C. Each pre-culture was separately inoculated into SD medium and aerobically cultivated for 24 h at 30 °C. Cells were collected and washed with distilled water. For xylose fermentation assay, these strains were inoculated into 50 ml fermentation medium, which contained 6.7 g/l of yeast nitrogen base without amino acids and 50 g/l of xylose. For glucose/xylose fermentation assay, these strains were inoculated into 50 ml fermentation medium, which contained 6.7 g/l of yeast nitrogen base without amino acids, 30 g/l of glucose, and 20 g/l of xylose. The initial cell density in the fermentation medium was adjusted to OD_600_ of 10. All fermentation experiments were performed at 30 °C with agitation (100 rpm).

Concentrations of glucose, xylose, glycerol, xylitol, and ethanol in the fermentation medium were analyzed by high-performance liquid chromatography (HPLC) (Prominence, Shimadzu, Japan) on a HPX-87H column (Bio-Rad), used together with a refractive index detector (model RID-10A, Shimadzu). The HPLC system was operated at 60 °C using 0.05% H_2_SO_4_ as the mobile phase (flow rate, 0.6 ml/min).

### Microaerobic fermentation

Microaerobic fermentations were carried out in 96-well Storage Blocks (Corning Incorporated, NY, USA). Yeast strains were aerobically pre-cultured in 96-well Storage Blocks (1 ml of SD medium per well), sealed with sterile breathable Nunc Sealing Tape (Thermo Fisher Scientific Inc.), with shaking at 1500 rpm in an M-BR-022UP constant temperature incubator shaker (Taitec Corporation) for 24 h at 30 °C. Next, 200 μl of the pre-culture was used to inoculate 1 ml of SD medium and cultured under the same condition for 24 h. Cells were harvested, washed twice with 1 ml of sterile water, and then suspended in 0.2 ml of sterile water to prepare yeast suspensions. Fermentation test was performed under the following conditions. One milliliter of SX medium, containing 20 g/l of xylose as the sole carbon source, was placed into each well of a 96-well storage block, to which yeast suspension was added to a final OD_600_ of 1.0 (for comparing xylose fermentation by the mutants generated by saturated mutagenesis) or to a final OD_600_ of 10.0 (for comparing xylose fermentation by other mutated XIs). For establishing a microaerobic condition, each well was hermetically sealed with Titer Stick HC film (Kajixx Co., Ltd., Japan), and fermentation was carried out in the M-BR-022UP incubator at 30 °C with shaking at 1500 rpm. An aliquot of the fermented liquid was taken out at different times as indicated and analyzed by HPLC.

### Introduction of mutation in RsXI-C1 and growth-based screening of mutants

To introduce random mutations in the XI genes, an error-prone PCR was carried out with GeneMorph II kit (Agilent technologies, CA, USA) using pRS436GAP-RsXIC1O as the template and primer pair pRSSacII-AAA-ATG-F4 and pRSXhoI-TAA-R3; sequences of these primers are listed in Additional file 10: Table S4. Using this method, we were able to introduce on average 3 mutations per 1000 bases in the DNA fragment (error rate 0.3%) by error-prone PCR.

A yeast centromeric vector pRS316GAP was constructed for introducing the mutated XI genes into yeast. The P_*TDH3*_-T_*CYC1*_ region was amplified from the plasmid pRS436GAP by PCR using the primers TDH3p-CYC1t-IF-F and TDH3p-CYC1t-IF-R (Additional file 10: Table S4). The amplified DNA fragment was inserted into the PvuII-digested pRS316 (NBRP Accession Number: BYP562) to generate pRS316GAP (Additional file 11: Figure S7e). In this plasmid, the cloned genes are under the control of the *TDH3* promoter and *CYC1* terminator.

The mutated *RsXI*-*C1O* library, produced by error-prone PCR (see above), was mixed with *Sac*II and *Xho*I digested pRS316GAP, and directly introduced into the W600W strain using the gap repair cloning protocol [48]. The resulting yeast transformants were cultured in SD medium for 2 days. The non-mutated DNA fragment (control), amplified by PCR using pRS436GAP-RsXIC1O as the template, was introduced into the W600W strain in the same way as described above.

One hundred microliter of the mutant library culture was added to 5 ml of SX medium, and incubated at 30 °C with shaking at 70 rpm in the Bio-photorecorder. After 7 days of cultivation, cells were harvested from the culture medium, and resuspended in 5 ml of fresh SX medium to a final OD_600_ of 0.1. After culturing for 7 days in the Bio-photorecorder, cells were recovered from the culture medium and then spread on an SX agar plate and incubated at 30 °C. Colonies growing faster than the control were selected and cultured on an SD agar plate. Twenty clones were selected for the growth experiment in SX medium, from which top 10 strains exhibiting better growth rates than the control strain were chosen for plasmid extraction. Plasmids were extracted using the Zymoprep Yeast Plasmid Minipreparation kit (Zymo research). DNA inserts of these extracted plasmids were sequenced completely to identify the introduced mutation. These plasmids were subsequently used to retransform W600W.

### Construction of the saturation mutagenesis library

An amino acid point mutation library, targeting the asparagine 337 (N337) residue of RsXI, was constructed by site-directed mutagenesis using the plasmid pRS316GAP-RsXIC1O as the template, primers listed in Additional file 10: Table S4, and a Quick Change Lightning MultiSite-Directed Mutagenesis kit (Agilent Technologies) following the protocol provided with the kit. Consequently, 18 plasmids, each expressing a different single amino acid substitution mutant (except asparagine and threonine), were obtained (Additional file 7: Table S3). Eighteen different strains were generated by transforming the W600W strain separately with each mutant plasmid. Resulting strains are listed in Additional file 2: Table S2.

### Introduction of mutation into other XIs

Preparation of DNA templates for introducing a point mutation was carried out as follows. The coding regions of *PiXIO* and *CpXIO* were amplified from the plasmids pRS436GAP-PiXIO and pRS436GAP-CpXIO, respectively, by PCR. The resulting DNA fragments were inserted into the *Sac*II/*Xho*I digested pRS316GAP plasmid to construct pRS316GAP-PiXIO and pRS316GAP-CpXIO, respectively. A synthetic *LlXI* (*LlXIO*), codons optimized for expression in yeast, was custom synthesized (GenScript Corporation); the amino acid sequence of LlXI was acquired from the GenBank (RefSeq Accession Number: WP_057720788.1). The resulting DNA fragment was cloned into the *Sac*II/*Xho*I digested pRS316GAP to construct pRS316GAP-LlXIO.

Plasmids pRS316GAP-PiXIO, pRS316GAP-CpXIO, and pRS316GAP-LlXIO were used as templates for the PCR-based site-directed mutagenesis. For each XI gene, four different single amino acid substitution mutants were created; thus, the respective asparagine residue in each XI was substituted with cysteine, threonine, valine, and alanine. Corresponding plasmids harboring these substitution mutants are listed in Additional file 7: Table S3. The resulting plasmids were individually introduced into the W600W strain to create the XI mutant expressing strains (Additional file 2: Table S2).

### Protein structure prediction

The three-dimensional predicted model of RsXI-C1 and N337C mutant of RsXI-C1 were generated based on the crystal structure of *Bacteroides thetaiotaomicron* xylose isomerase (Protein Data Bank code 4XKM) [49] using Discovery Studio (Dassault Systèmes, France).

## Additional files

**Additional file 1: Table S1.** Identities between novel XIs and other functional XIs.

**Additional file 2: Table S2.** List of strains.

**Additional file 3: Figure S1.** RT-PCR analysis of fractionated symbiotic bacteria and protists. Symbiotic bacteria and protists in termite hindgut were fractionated by slow centrifugation and each fraction was used for RT-PCR analysis as described in "Methods". Amplified products were analyzed by electrophoresis on 1% agarose gel. Left lane, standard DNA markers: 23,130 bp, 9416 bp, 6557 bp, 4361 bp, 2322 bp, 2027 bp, 1353 bp, 1078 bp, 872 bp, and 603 bp. Arrowhead, amplified fragment from the *RsXI*-*C1.*

**Additional file 4: Figure S2.** Microaerobic xylose fermentation by recombinant PP600 strains. *S. cerevisiae* PP600 expressing RsXI-C1 (MR311), PiXI (MP111), and CpXI (MC111) were cultivated under microaerobic fermentation condition in SX medium as described in "Methods". The initial cell density was same (OD_600_ = 5) in all cases. Amount of xylose consumed by each strain was determined by HPLC. Xylose consumed after 120 h fermentation was shown. Error bars represent standard deviations of biological tetraplicates. Statistical significance was determined using Student’s *t* test. *P < 0.05, significant difference.

**Additional file 5: Figure S3.** Relative expression of xylose utilization related genes in recombinant yeast strains. Recombinant strains (WR311, WP111, WC111, and WVC110) were aerobically pre-cultivated in SD medium at 30 °C for 24 h. Each pre-culture was separately inoculated into SD medium. The initial cell density was adjusted to OD_600_ of 0.05, and aerobically cultivated at 30 °C. After 24 h cultivation, cells were lysed and total RNA was extracted using High Pure RNA Isolation Kit (Roche, Switzerland) according to the manufacturer’s instructions. Reverse transcription of extracted RNA was carried out using high capacity RNA-to-cDNA Kit (Thermo Fisher Scientific Inc.) according to the manufacturer’s instructions. Quantitative PCR was carried out using a qPCR detection system (ABI PRISM 7000 sequence detection system, Thermo Fisher Scientific Inc.) and power SYBR Green Master Mix (Thermo Fisher Scientific Inc.). Primer sequences used in this experiment were listed in Additional file 10: Table S4. Relative gene expression values were calculated by the &&CT method and normalized by housekeeping gene *TUB6*. Error bars represent standard deviations of biological triplicates. *XKS1*, xylulokinase; *TKL1*, transketolase 1; *TKL2*, transketolase 2; *TAL1*, transaldolase; *RKI1*, ribose 5-phosphate isomerase; *RPE1*, ribulose 5-phosphate epimerase; *GND2*, 6-phosphogluconate dehydrogenase; *SOL4*, 6-phosphogluconolactonase; *ZWF1*, glucose-6-phosphate dehydrogenase; *HXT1*, hexose transporter 1; *HXT5*, hexose transporter 5; *HXT7*, hexose transporter 7; *GAL2*, : galactose permease.

**Additional file 6: Figure S4.** Specific growth rates of mutant strains isolated from growth-based screening on xylose. Growth assays in liquid culture of 20 mutant strains were carried out in SX medium under aerobic condition. Growth rates were determined as described in "Methods".

**Additional file 7: Table S3.** List of plasmids.

**Additional file 8: Figure S5.** Xylose fermentation by recombinant W600W strains. *S. cerevisiae* W600W expressing RsXI-C1 (WR320), PiXI (WP120), LlXI (WL120), and CpXI (WC120) were cultivated under microaerobic fermentation condition in SX medium as described in "Methods". The initial cell density was same (OD_600_ = 10) in all cases. Amount of xylose consumed by each strain was determined by HPLC. Xylose consumed after 72 h fermentation was shown. Error bars represent standard deviations of biological duplicates.

**Additional file 9: Figure S6.** Predicted structures of the active sites of RsXI-C1 and N337C mutant of RsXI-C1. Predicted three-dimensional structures of the active sites of (**a**) wild-type RsXI-C1 and (**b**) N337C mutant of RsXI-C1 (see text for the details regarding model building). Positions of the Asn337 (wild-type) and Cys337 (N337C mutant) residues are indicated. Also shown are the active site residues (Phe101, His102, Asp103, and Lys235), metal ion binding residues (Glu233, Glu269, Asp297, and Asp339), and manganese ion (blue sphere).

**Additional file 10: Table S4.** Primers used in this study.

**Additional file 11: Figure S7.** Maps of plasmid vectors used in this study. (**a**) The multicopy plasmid for the expression of XI genes. (**b**) The low-copy centromeric plasmid for the expression of XI genes. (**c**) The integration plasmid targeted to the HIS3 loci in chromosome XV for the expression of *XKS1*. (**d**) The integration plasmid targeted to the upstream region of *ADH3* in chromosome XIII for the expression of *TAL1* and *TKL1*. (**e**) The integration plasmid targeted to the *GRE3* loci in chromosome VIII for the expression of *RPE1* and *RKI1.*

## Abbreviations

XI: xylose isomerase
XR: xylose reductase
XDH: xylitol dehydrogenase
XK: xylulokinase
PPP: pentose phosphate pathway
PiXI: XI from *Piromyces* sp. E2
RsXI: XI from the protists in the hindgut of *Reticulitermes speratus*
CpXI: XI from *Clostridium phytofermentans*
LlXI: XI from *Lactococcus lactis*
HPLC: high-performance liquid chromatography
OD_600_: optical density at 600 nm
OD_660_: optical density at 660 nm
