# Supplementary figures and images for: Screening and evolution of a novel protist xylose isomerase from the termite Reticulitermes speratus for efficient xylose fermentation in Saccharomyces cerevisiae
Source: Biotechnol Biofuels. 2017 Aug 23;10:203. doi: 10.1186/s13068-017-0890-1 (PMC5569483; doi:10.1186/s13068-017-0890-1)

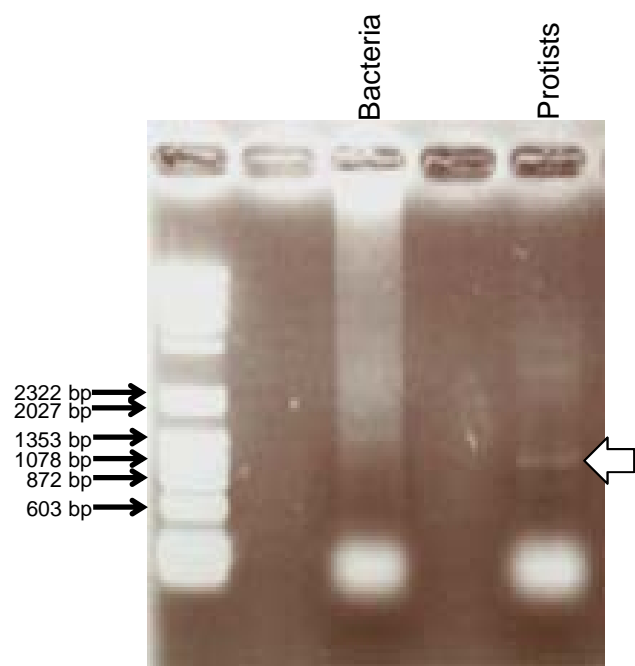

Supplement: Supplementary file 3 — Additional file 3: Figure S1. RT-PCR analysis of fractionated symbiotic bacteria and protists. Symbiotic bacteria and protists in termite hindgut were fractionated by slow centrifugation and each fraction was used for RT-PCR analysis as described in "Methods". Amplified products were analyzed by electrophoresis on 1% agarose gel. Left lane, standard DNA markers: 23,130 bp, 9416 bp, 6557 bp, 4361 bp, 2322 bp, 2027 bp, 1353 bp, 1078 bp, 872 bp, and 603 bp. Arrowhead, amplified fragment from the RsXI-C1. [file 13068_2017_890_MOESM3_ESM.pdf]

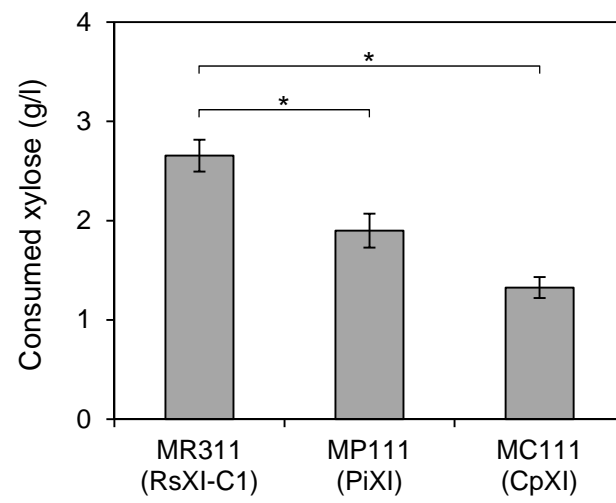

Supplement: Supplementary file 4 — Additional file 4: Figure S2. Microaerobic xylose fermentation by recombinant PP600 strains. S. cerevisiae PP600 expressing RsXI-C1 (MR311), PiXI (MP111), and CpXI (MC111) were cultivated under microaerobic fermentation condition in SX medium as described in "Methods". The initial cell density was same (OD600 = 5) in all cases. Amount of xylose consumed by each strain was determined by HPLC. Xylose consumed after 120 h fermentation was shown. Error bars represent standard deviations of biological tetraplicates. Statistical significance was determined using Student’s t test. *P < 0.05, significant difference. [file 13068_2017_890_MOESM4_ESM.pdf]

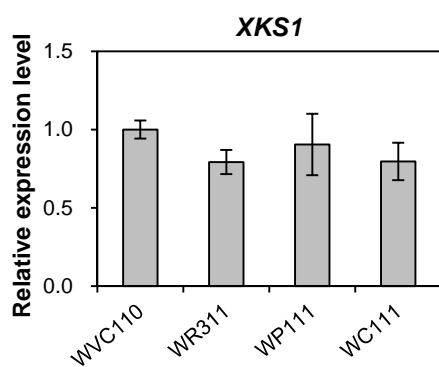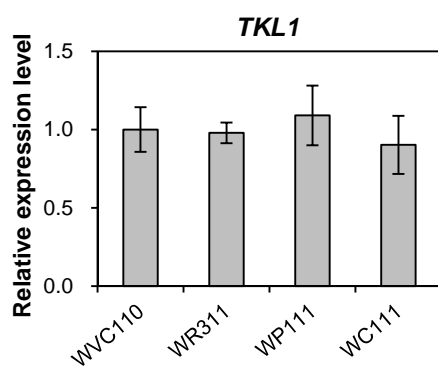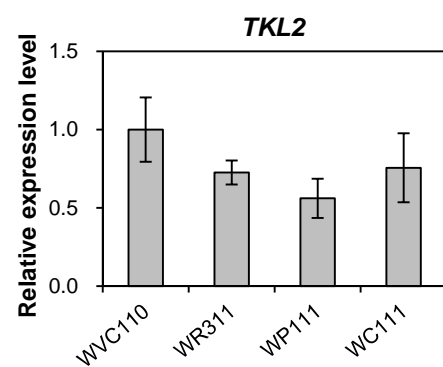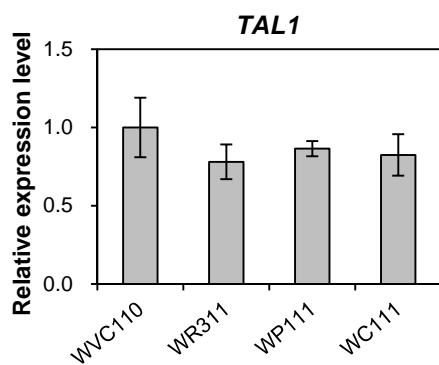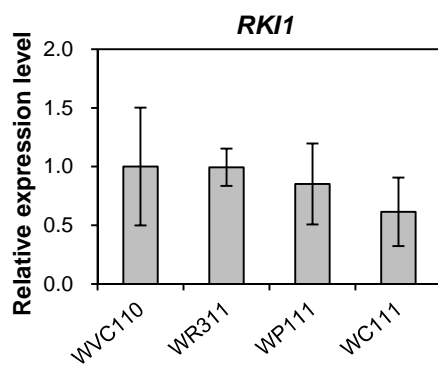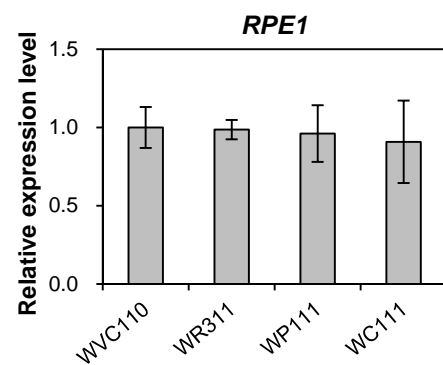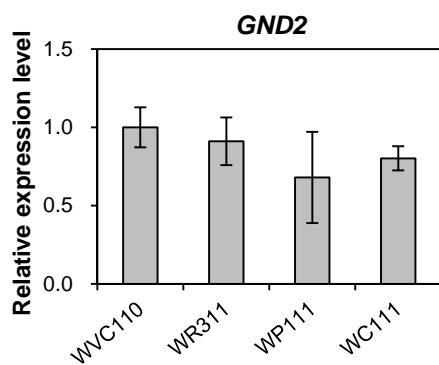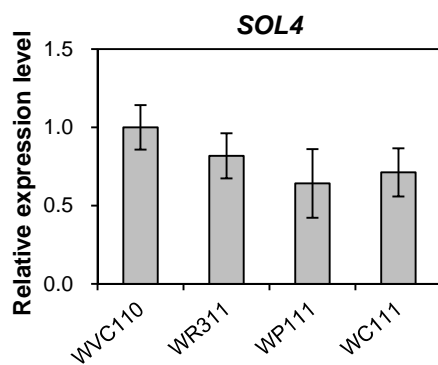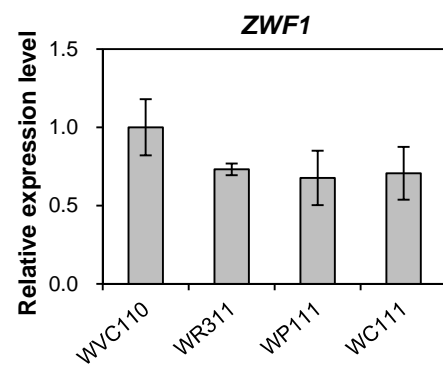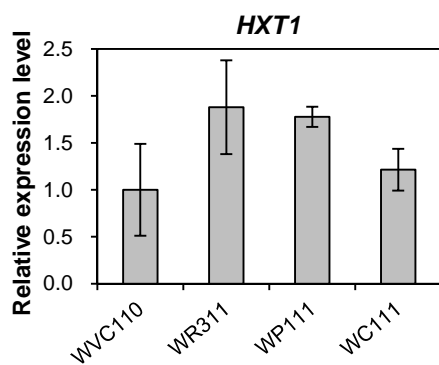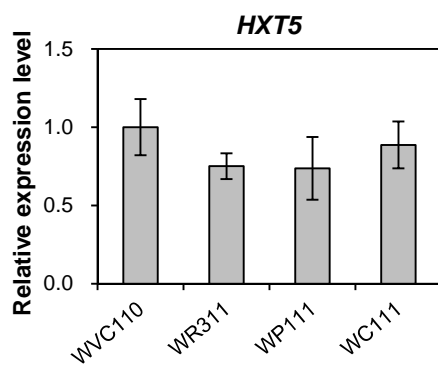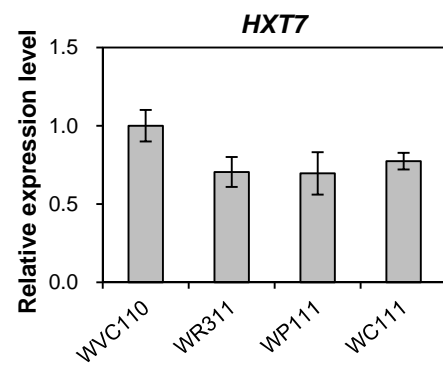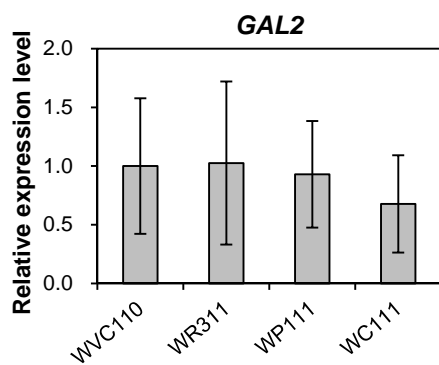

Supplement: Supplementary file 5 — Additional file 5: Figure S3. Relative expression of xylose utilization related genes in recombinant yeast strains. Recombinant strains (WR311, WP111, WC111, and WVC110) were aerobically pre-cultivated in SD medium at 30 °C for 24 h. Each pre-culture was separately inoculated into SD medium. The initial cell density was adjusted to OD600 of 0.05, and aerobically cultivated at 30 °C. After 24 h cultivation, cells were lysed and total RNA was extracted using High Pure RNA Isolation Kit (Roche, Switzerland) according to the manufacturer’s instructions. Reverse transcription of extracted RNA was carried out using high capacity RNA-to-cDNA Kit (Thermo Fisher Scientific Inc.) according to the manufacturer’s instructions. Quantitative PCR was carried out using a qPCR detection system (ABI PRISM 7000 sequence detection system, Thermo Fisher Scientific Inc.) and power SYBR Green Master Mix (Thermo Fisher Scientific Inc.). Primer sequences used in this experiment were listed in Additional file 10: Table S4. Relative gene expression values were calculated by the &&CT method and normalized by housekeeping gene TUB6. Error bars represent standard deviations of biological triplicates. XKS1, xylulokinase; TKL1, transketolase 1; TKL2, transketolase 2; TAL1, transaldolase; RKI1, ribose 5-phosphate isomerase; RPE1, ribulose 5-phosphate epimerase; GND2, 6-phosphogluconate dehydrogenase; SOL4, 6-phosphogluconolactonase; ZWF1, glucose-6-phosphate dehydrogenase; HXT1, hexose transporter 1; HXT5, hexose transporter 5; HXT7, hexose transporter 7; GAL2, : galactose permease. [file 13068_2017_890_MOESM5_ESM.pdf]

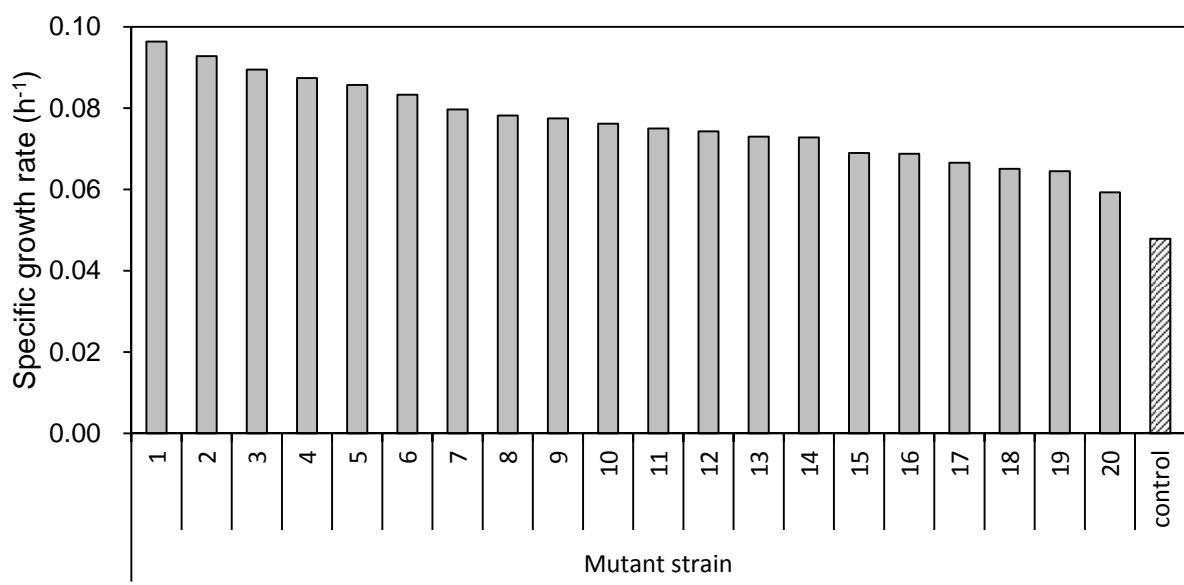

Supplement: Supplementary file 6 — Additional file 6: Figure S4. Specific growth rates of mutant strains isolated from growth-based screening on xylose. Growth assays in liquid culture of 20 mutant strains were carried out in SX medium under aerobic condition. Growth rates were determined as described in "Methods". [file 13068_2017_890_MOESM6_ESM.pdf]

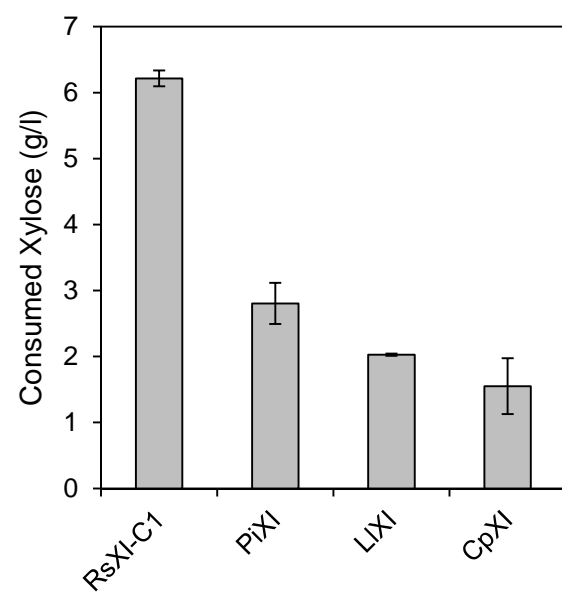

Supplement: Supplementary file 8 — Additional file 8: Figure S5. Xylose fermentation by recombinant W600W strains. S. cerevisiae W600W expressing RsXI-C1 (WR320), PiXI (WP120), LlXI (WL120), and CpXI (WC120) were cultivated under microaerobic fermentation condition in SX medium as described in "Methods". The initial cell density was same (OD600 = 10) in all cases. Amount of xylose consumed by each strain was determined by HPLC. Xylose consumed after 72 h fermentation was shown. Error bars represent standard deviations of biological duplicates. [file 13068_2017_890_MOESM8_ESM.pdf]

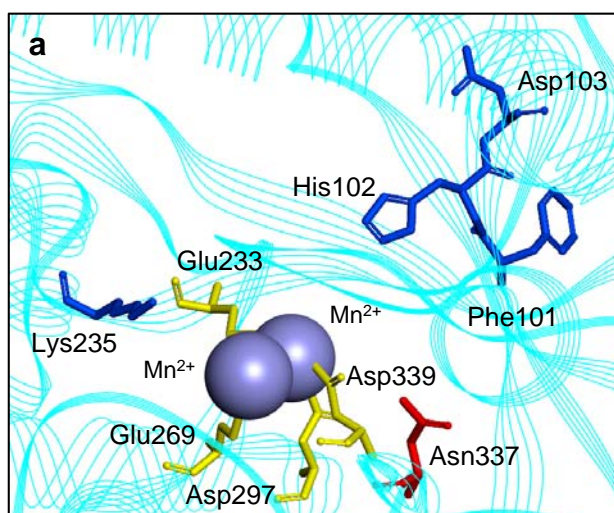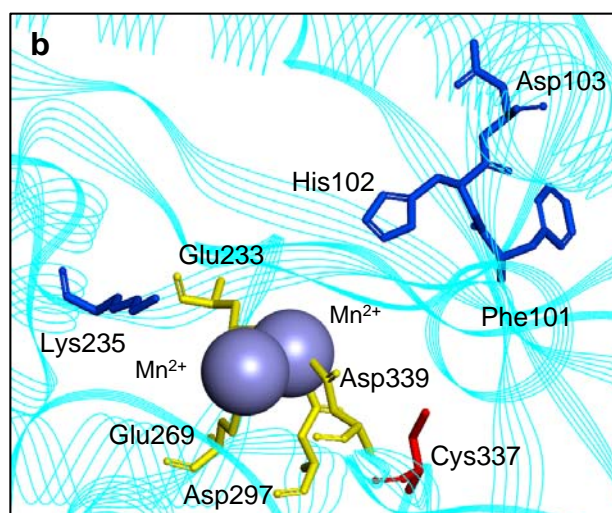

Supplement: Supplementary file 9 — Additional file 9: Figure S6. Predicted structures of the active sites of RsXI-C1 and N337C mutant of RsXI-C1. Predicted three-dimensional structures of the active sites of (a) wild-type RsXI-C1 and (b) N337C mutant of RsXI-C1 (see text for the details regarding model building). Positions of the Asn337 (wild-type) and Cys337 (N337C mutant) residues are indicated. Also shown are the active site residues (Phe101, His102, Asp103, and Lys235), metal ion binding residues (Glu233, Glu269, Asp297, and Asp339), and manganese ion (blue sphere). [file 13068_2017_890_MOESM9_ESM.pdf]

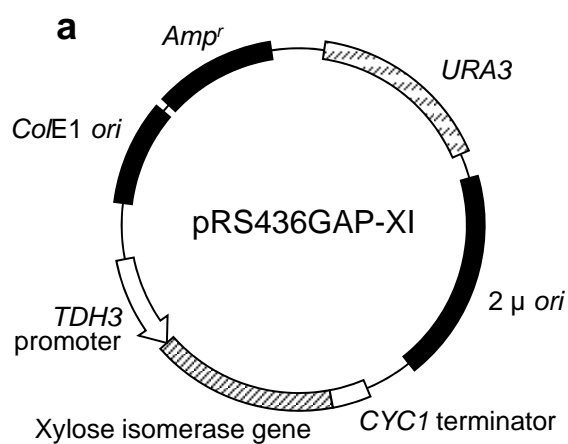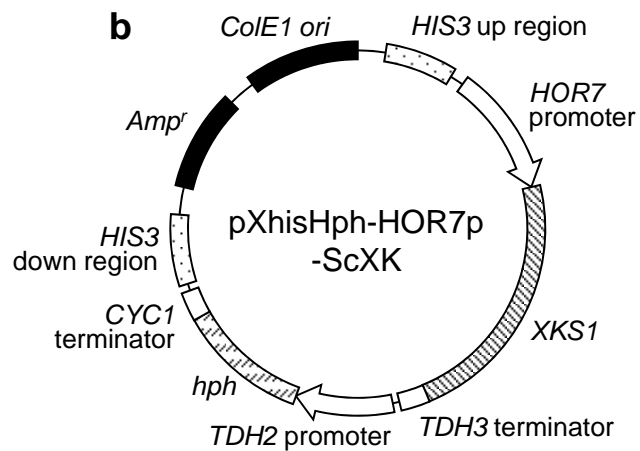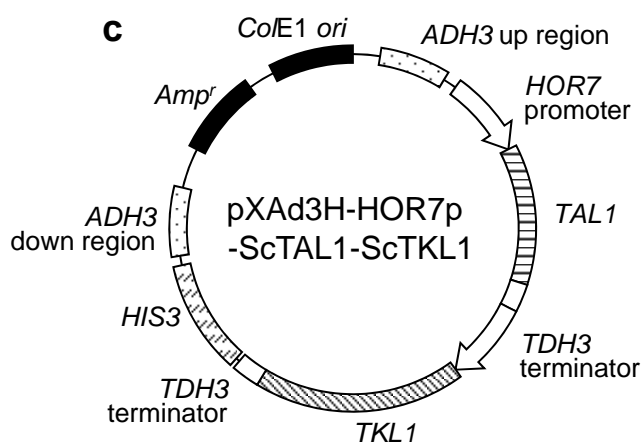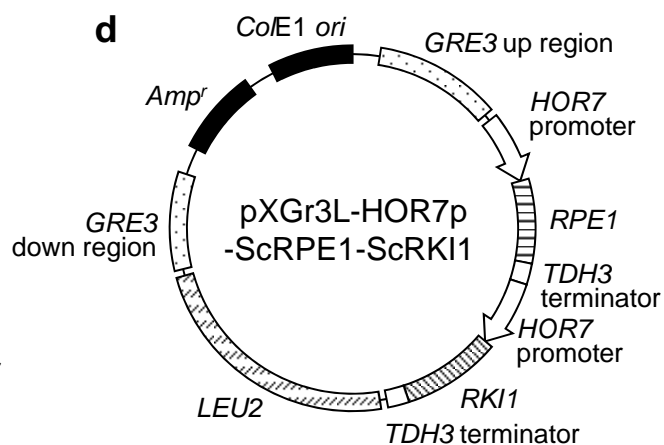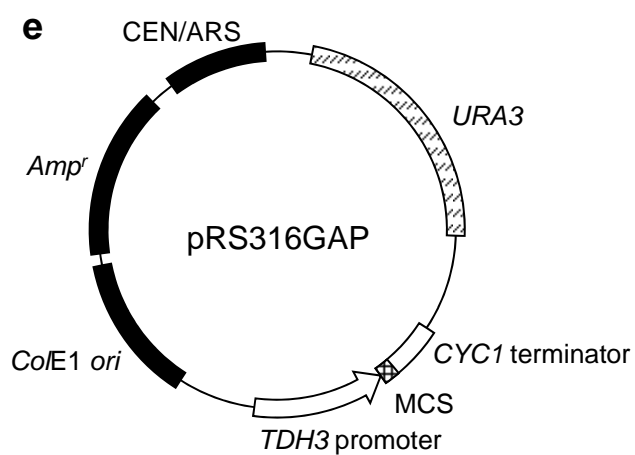

Supplement: Supplementary file 11 — Additional file 11: Figure S7. Maps of plasmid vectors used in this study. (a) The multicopy plasmid for the expression of XI genes. (b) The low-copy centromeric plasmid for the expression of XI genes. (c) The integration plasmid targeted to the HIS3 loci in chromosome XV for the expression of XKS1. (d) The integration plasmid targeted to the upstream region of ADH3 in chromosome XIII for the expression of TAL1 and TKL1. (e) The integration plasmid targeted to the GRE3 loci in chromosome VIII for the expression of RPE1 and RKI1. [file 13068_2017_890_MOESM11_ESM.pdf]
